# Nasal Gel Loaded with Amphotericin Nanotransferosomes as Antifungal Treatment for Fungal Sinusitis

**DOI:** 10.3390/pharmaceutics13010035

**Published:** 2020-12-28

**Authors:** Khaled M. Hosny, Nabil A. Alhakamy

**Affiliations:** 1 Department of Pharmaceutics, Faculty of Pharmacy, King Abdulaziz University, Jeddah 21589, Saudi Arabia; nalhakamy@kau.edu.sa; 2 Center of Excellence for Drug Research and Pharmaceutical Industries, King Abdulaziz University, Jeddah 21589, Saudi Arabia

**Keywords:** Amphotericin B, Box-Behnken Design, clove oil, *Aspergillus flavus*, nanotransferosomes

## Abstract

On the basis of fungal involvement, rhinosinusitis is categorized into allergic, mycetoma, chronic, and acute invasive types. The aim of the current study was to evaluate the efficacy of an amphotericin gel in situ loaded with nanotransferosomes against *Aspergillus flavus*, which causes allergic rhinosinusitis. A Box–Behnken design was utilized to study the interaction among the nanotransferosomes and optimize independent variables in formulating them, in order to match the prerequisites of selected responses. The optimal formulation was determined to be 300 mg/mL soybean lecithin, 200 mg/mL amphotericin B (AMP), and 150 mg/mL clove oil, resulting in a particle size of 155.09 nm, 84.30% entrapment efficacy (EE), inhibition zone of 16.0 mm, and 0.1197 mmol serum creatinine. The optimized batch was further prepared into an in situ gel and evaluated for various parameters. The optimized formulation released 79.25% AMP and enhanced permeation through the nasal membrane, while the other formulations did not achieve complete absorption. According to in vivo tests using rabbits as animal models, the optimized AMP-nanotransferosomal formulations (NT) in in situ gel result in a non-significant difference among the various kidney function parameters. In conclusion, nasal in situ gel loaded with AMP-clove oil nanotreansfersomes can act as a promising novel carrier that enhances antifungal activity and decreases AMP nephrotoxicity.

## 1. Introduction

In recent years, an escalating number of paranasal sinus fungal infections has been reported. The majority are attributed to the *Aspergillus* species [1,2], while others include other fungal pathogens of the *Zygomycetous* and *Dematiaceous* genera [3]. On the basis of pathological conditions, fungal rhinosinusitis is characterized into non-invasive (allergic and mycetoma) and invasive (chronic and acute) types [4,5].

Amphotericin B (AMP) is a broad spectrum antibiotic that has been widely used to treat fungal infections (as a first-line management) and various leishmaniasis (as a second line management) [6,7]. Since its discovery in the 1960s, various dosages and formulations have been researched in order to lessen the toxicity and augment the therapeutic efficiency. In particular, several nanoparticle formulations, including polymeric nanoparticles [8,9], nanocapsule [10], liposome [11], solid lipid nanoparticle [12,13] and micelles [14], have been developed to achieve the foresaid goals. Several commercial forms of AMP, such as micellar dispersion, liposomes, lipid-base formulation, have been also been intensively researched. However, these systems suffer from dose-dependent toxicity [15], shortage of support for long-standing benefits [16,17], towering expenditure, and contradictory results. Furthermore, AMP possesses several intrinsic confines, such as propensity to degradation (oxidation and/or light induced), low water-solubility, hepatotoxicity, nephrotoxicity, in addition to anemia-related symptoms [18]. Thus, numerous investigations have been performed on several carriers for AMP as a protection mechanism from various degradation pathways and to reduce its inherent toxicity.

Eugenol, a chief active ingredient in clove oil, has proven analgesic, anti-inflammatory antibacterial, and local anesthetic effects. In addition, eugenol exhibits anti-inflammatory activity through a cyclooxygenase II inhibitor [COX-2 Inhibitor], as proposed in molecular studies by Hong et al. [19]. Also, Jadhav et al. suggested the anti-inflammatory activity and analgesic activity of eugenol through cycloxygenase-II enzyme and capsaicin receptor activity, respectively [20].

Other studies report that eugenol has antibacterial activity against several Gram positive and negative bacteria and can be used in a topical application with prilocaine or lidocaine owing to its anti-nociceptive action. Eugenol at a concentration of 5–18% is safe for topical application; it may cause local irritation when present in concentration more than 18% [21].

Drug delivery through the skin for systemic benefits has been realized by a wide range of transdermal systems, but still faces several issues related to the permeation of active moieties. Therefore, various nano/vesicular systems have been shown to be a promising alternative to enhance permeation across the skin [22]. In this view, a new type of carrier system called “transferosome” has been introduced. Transferosomes are ultra-flexible lipid molecular aggregates consisting of an inner aqueous section bounded by lipid bilayer and, thus, are efficient in delivering both low and high molecular weight drugs. The lipid layer allows special customized properties, which are attributable to the occurrence of “edge activators” [23]. When applied under non-occlusive conditions, the flexibility of nano-transferosomes minimizes the risk of vesicle rupture and follows a natural water gradient pathway. Nano-transferosomes can penetrate through the stratum corneum via trans and inter cellular routes, and trans-pore hydrostatic pressure difference serves as the driving force for this transportation [24].

Response surface methodology (RSM) is the most accepted strategy for the development and optimization of several drug-delivery systems [25]. RSM involves the application of various experimental designs and polynomial relationships to map the responses over the experimental domain. Different types of design, such as D-Optimal, central composite, and Box–Behnken designs (BBD), are available for statistical optimization. For the current study, BBD was selected due to its independent, rotatable designs for treatment combinations, fewer experimental runs, and cost-effective technique in optimizing the formulation preparation [26].

The rationale for selecting the nano-transferosomes was to attain to enhance the solubility and attain optimal delivery of the selected AMP (low-soluble drug) following an invasive approach.

The impact of eugenol as edge activator and to enhance the anti-microbial activity of AMP was studied through RSM. The objective of the present work was to design and formulate an in situ gel loaded with the optimized formulation of nano-transferosomes using ellagic acid to improve the bioavailability and diminish the dose-related toxicities.

## 2. Materials and Methods

### 2.1. Materials

Amphotericin B was purchased from Sigma-Aldrich Co. (St Louis, MO, USA). Tween was acquired as a generous gift from Saudi Drugs and Medical Instruments Company (SPIMACO), in Qassim, Saudi Arabia. Clove oil was procured from Avanti Polar liquids (Alabaster, AL, USA). High-performance liquid chromatography (HPLC) grade solvents were collected form Merck (Germany). All other reagents and chemicals used were of analytical grade.

### 2.2. Methods

#### 2.2.1. Preparation of Amphotericin B Nanotransferosomes (AMP-NT)

A conventional thin-layer evaporation technique was utilized to prepare AMP-loaded nanotransferosomal formulations (AMP-NT). The effect of the selected independent variables on different responses was demonstrated by applying 3 factor, 3 level BBD. As per the projected experimental plan (Table 1), a total of 17 formulations were planned per the design. Specific amounts of soybean, AMP, and clove oil were added to a dried round-bottom flask containing the solvent mixture (methanol and chloroform at 2:1 *v*/*v*) and allowed to evaporate at 45 °C, at 60 rpm under low pressure Heidolph rotavap ML/G3 (Heidolph Instruments GmbH& CO., Schwabach, Germany) until a thin lipid layer formed on the inner walls of the flask. The final solvent residuals were removed under vacuum. Finally, the dried thin layer was reconstituted with PBS (phosphate buffer) with pH of 7.4 under continuous stirring for 60 min. The vesicles formed were allowed to swell at room temperature for 2–3 h [27,28]. The multilamellar vesicles were sonicated by Digital Sonifier (St Louis, MO, USA) for 15–20 min to lessen the vesicles’ size and subsequently stored at 4 °C until further study.

#### 2.2.2. Experimental Design

BBD was performed using DESIGN EXPERT 12 (Stat-Ease Inc., Minneapolis MN, USA) to study the interaction effects. Furthermore, factor association between the variables was analyzed by response surface graphs. Soybean lecithin (mcg/mL) (X1), AMP (mcg/mL) (X2), and clove oil (mcg/mL) (X3) were considered the independent variables, and the factorial levels for these factors were coded as −1 (low level), 0 (medium level), or +1 (high level) [23]. Particle size (PS-Y1), entrapment efficacy (EE-Y2), inhibition zone (Y3) and serum creatinine (Y4) were chosen as the responses to be evaluated. Analysis of variance (ANOVA) was requisite to ascertain the statistical rationale of generated equations [29,30]. All experimental results were concomitantly fitted to various models, and the best fitting models (main, interaction, or quadratic) were selected based on statistical parameters, including multiple correlation coefficient (R2), adjusted R2, and predicted R2. In general, the non-linear quadratic design matrix is defined as follows:

(1) $$Y_{i\left(Quadratic\right)}=b_{0}+b_{1}X_{1}+b_{2}X_{2}+b_{3}X_{3}+b_{12}X_{1}X_{2}+b_{13}X_{1}X_{3}+b_{23}X_{2}X_{3}+b_{11}X_{1}^{2}+b_{22}X_{2}^{2}+b_{33}X_{3}^{2}$$

#### 2.2.3. Characterization of AMP-NT

AMP-NTs were characterized for vesicle size and zeta potential using (Malvern instruments, Malvern, UK) by a dynamic light scattering process [31]. Each formulation was measured three times (*n* = 3) to obtain averages [32].

#### 2.2.4. Entrapment Efficacy (*EE*)%

*EE* was generally expressed as a fraction of drug available in the prepared AMP-NT and was determined by an indirect technique. The prepared formulation was added to a Petri dish. Freeze-dried, then the required quantity of acetonitrile was added and mixed vigorously to the dried sample [27]. The resulting dispersion was then centrifuged for 1 h at 10,000 rpm. The supernatant liquid formed was collected and then washed again with acetonitrile. All of the washing contents and supernatant liquid were added together and dried using a water bath. Subsequently, a precise amount of methanol was added to the final dried extract, which was diluted and measured at an absorbance of 450 nm. *EE* was estimated using the following formula:

(2) $$EE\;\left(\%\right)=\frac{Ctotal\;-\;Cfree}{Ctotal}$$

where *Ctotal* is the theoretical concentration; and *Cfree* is the concentration of drug found in the supernatants.

### 2.3. In Situ Gel Preparation

In situ gel formulation of AMP was prepared by sprinkling Deacetylated gellan gum DCG (cationic induced in situ gel polymer) into 6 mL distilled water at high temperatures (80 ± 2 °C), then the solution was stirred continuously until the polymer dissolved. The dispersion formed was allowed to cool throughout the night [33,34]. Another 4 mL AMP-NT was added to the above prepared solution and then mixed to produce the situ gel of AMP.

### 2.4. Evaluation of AMP-NT In Situ Gels

#### 2.4.1. Critical Ionic Concentration (CIC)

The critical ionic concentration (CIC) for phase transition is an imperative parameter for in situ gels that are activated through ions. Initially, various quantities of artificial nasal fluid (NF) and 1 mL of 0.5% Deacetylated gellan gum solution were mixed together. Then, the resulting solution was inserted into bottles and placed in a water bath maintained at a temperature of 32 °C. After 20 s, the filled bottles were turned over. If the formulation contains the gel formation, then the content will adhere to the bottom of the bottle instead of sliding or flowing down; this occurrence is noted as “+”. The least amount of NF concentration that could persuade the gel formation was projected as CIC [35].

#### 2.4.2. Expansion Coefficient (S%)

As per the nature of in situ gels, as a solution transforms into a gel, its volume may expand. This may cause uneasiness in the nasal cavity, owing to its small size. Thus, the expansion coefficient, S%, was evaluated by mixing 1 mL DGG solution (0.5%) and 0.25 mL NF in a graduated test tube and kept in a water bath at 32 °C (The initial volume V_I_ = 1.25 mL). About 2 mL of the optimized formulation was added to the above solution. The Final volume (V_F_ = 3.25), and changes in the volume after gelation (V_G_) were noted [36]. S% was calculated using the following formula:

(S%) = (V_G_ − V_I_)/V_I_ × 100. (3)

#### 2.4.3. Rheological Properties

The apparent viscosity values of the optimized formulation were obtained before and after gelation (after 30 s) using a Brookfield Digital Viscometer at 10 rpm.

#### 2.4.4. Gel Strength Measurement

Gel strength is an indirect sign of the viscosity of a prepared formulation under physiological conditions. After NF was added (neutralization reaction), about 5 g formulation transformed into a gel. Subsequently, 3.5 g formulation was positioned on the top of the in situ gel obtained, and the time required for the formulation to reach a depth of 3 mm in the gel was noted as the gel strength.

### 2.5. In Vitro Drug Release Studies

AMP release from the optimized formulation was determined by using a dialysis bag method. Around 5 mL formulation was placed into the dialysis bag and then engrossed in a vessel containing 100 mL phosphate buffer solution (pH = 6) at 37 ± 1 °C at 50 rpm. Samples were withdrawn for 12 h at regular intervals of time and analyzed for drug content.

### 2.6. Ex Vivo Permeation Studies

Fresh nasal tissue carefully collected from goat nasal cavities was provided by a local slaughterhouse. Collected tissue (area of 1.76 cm^2^) was mounted onto a Franz diffusion cell, and 7 mL phosphate buffer (pH = 6) was filled into the receptor chamber at 37 ± 1 °C. Pre-incubation time was maintained around 20 min [37]. After this period, 1 g of the optimized formulation and 1 mL pure drug suspension were placed into the donor chamber. At regular intervals of time, 0.5 mL sample was withdrawn, replaced with 0.5 mL fresh phosphate buffer (pH = 6), and the drug content in the sample was measured using high-performance liquid chromatography (HPLC) with a column: Phenomenex luna C18 (250 × 4.6 mm, 5 μm); mobile phase: 55/45 organic phase (41/18/10 methanol/acetonitrile/tetrahydrofuran)–buffer (2.5 mmol L^−1^ Ethylene-diamine-tetracetic acidEDTA-2Na); flow rate: 1.0 mL min^−1^; column temperature: 30 °C; detection wavelengths: 383 and 303 nm; injection volume: 20 μL. Several skin permeation parameters, including the diffusion coefficient (D), enhancement ratio (ER), permeability coefficient and steady state transdermal flux (Jss), were calculated from the permeation data obtained.

### 2.7. Nephrotoxicity Studies

**Figure 1:**
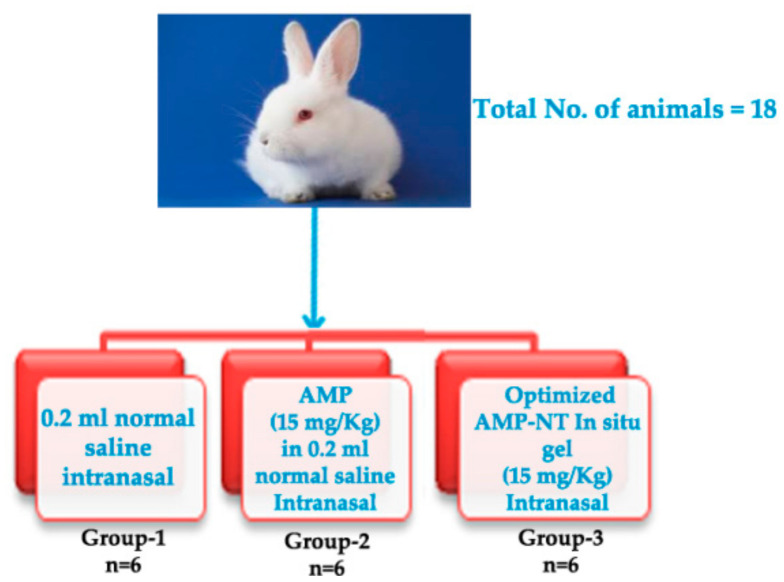
In vivo animal study groups and sample information.

A total of 18 rabbits were acquired from the Beni-Suef Clinical laboratory center, Beni-Suef. Egypt. All conventions were affirmed by the Animal Ethics Committee of the Beni-Suef Clinical Laboratory (Approval NO. 1-02-18, at Jan. 2018) and complied with the Declaration of Helsinki. Grouping of animals and the sample feed are depicted in Figure 1 [38]. Initially, all the acquired animals were adapted at 20 ± 1 °C for no less than 14 days under natural climatic conditions (12/12 h dark/light cycle) with free admittance to water and feed.

## 3. Results and Discussion

### 3.1. Optimization of AMP-NT

A Box–Behnken design with mixture design was used to explore the impact of the selected variables on minimum particle size, serum creatinine (Y1 and Y4), maximum EE, and inhibition zone (Y2 and Y3), [39]. A total of 19 runs were conducted, and the evaluated responses are presented in Table 2. The particle size of all trial batches was found to be in the range of 73 to 210 nm, and EE and inhibition zone were estimated in the range of 58–89% and 4–28 mm, respectively. The results obtained were statistically evaluated to determine individual responses to the selected variables by applying analysis of variance (ANOVA) and the fx model.

Different models were prepared according to the fit summary of responses (adjusted and predicted R2) and sequential sum of squares (Type-I), as shown in Table 3. In these models, the selected parameters were not aliased in order to find the highest order polynomial [40]. The precision of the models was further studied using the normal probability of studentized residuals, which were those that were scattered closest to the straight line with a slight deviation. ANOVA was performed to further evaluate the quantitative association between the responses and variables.

### 3.2. Response I

Among the applied models, the quadratic model exhibited the maximum Adju. R2 (0.9936) and Pred. R2 (0.9715) values and, thus, was chosen over the linear, 2FI, and cubic models. Furthermore, this was supported by a significant model F-value (41.64) and there is only 0.01% chance that an F-value (6.40) this large could occur due to noise. A non-significant lack of fit with a *p* value of 0.2969 suggests that the model is efficient. The coefficient of variation (CV), which helps to determine the reproducibility of the model, was found to be <10% (2.42%).

Additionally, adequate precision is determined according to the signal to noise ratio, where a ratio larger than 4 is desired. Herein, the ratio of 54.133 obtained confirms the ample signal to navigate the design space [41].

The polynomial equation can be further applied to predict the response from any given concentrations of independent variables and generate the relation using multiple regression analysis. From this, the response for any given level of selected factors can be predicted to identify the relative impact between them by comparing the coefficients.

ANOVA results revealed the significant statistical relationship between the components and responses at a 95% confidence level. Both *p* values of ANOVA and polynomial equations were used to estimate the true effect of variables, where *p* values less than 0.0500 indicate that the model terms are significant. According to the ANOVA results, Response I was significantly affected by three independent factors via a synergistic effect (*p*-value of <0.0001) [42,43] with A having highest magnitude of 38.46 (Table 4); thus, factors C and the polynomial terms of A affect the response antagonistically. The final equations in terms of the coded factors was determined to be:

Particle size = +155.09 + 38.46 A + 28.44 B − 4.58 C + 8.44 AB + 0.9412 AC − 0.0588 BC − 22.28 A^2^ + 0.6754 B^2^ − 1.19 C^2^ (4)

### 3.3. Response II

The quadratic model was selected for Response II on basis of the maximum Adju. R2 (0.9967) and Pred. R2 (0.9911) values in comparison to the other models. The model F-value (598.28) was found to be significant with only a 0.01% chance the large F-value is due to noise. The CV value was found to be <10% (0.8142%), thus confirming the reproducibility of the model. The adequate precision ratio of 74.659 indicates an ample signal to navigate the design space [41].

Both *p* values of ANOVA and polynomial equations were used to estimate the true effect of variables. Per ANOVA results, Response II was affected significantly by A and C factors via a synergistic effect (*p* value < 0.0001) [39,40] with A having the highest magnitude of 38.46 (Table 4), while factors B and the polynomial terms of B affect the response antagonistically. The final equation in terms of coded factors is:

*EE*% = +84.30 + 4.23 A − 3.46 B + 1.55 C − 0.2791 AB − 0.5291 AC − 0.0291 BC − 0.5952 A^2^ − 19.08 B^2^ − 0.2263 C^2^ (5)

### 3.4. Response III

Maximum Adju. R2 of 0.9978 and Pred. R2 of 0.9935 were determined for the quadratic model or Response I. The highest model F-value of 915.93 indicates that there is only a 0.01% chance that an F-value this large could be due to noise. As required, the CV value of 2.51% confirms the reproducibility of the selected model. Both *p* values of ANOVA and polynomial equations were used to estimate the true effect of the variables, which reveal that Response III was significantly affected by B, C, and BC factors via synergistic effect (*p* value < 0.0001) with the highest magnitude of 38.46 for factor A (Table 4). Factors A and polynomial terms of B and C affect the response antagonistically. The final equation in terms of coded factors is:

Inhibition zone = +16.09 − 0.7066 A + 9.67 B + 2.21 C − 0.0588 AB − 0.0588 AC + 0.6912 BC − 0.1125 A^2^ − 1.49 B^2^ + 0.5583 C^2^ (6)

### 3.5. Response IV

The model F-value of 367.49, maximum Adju. R2 of 0.9946, and Pred. R2 of 0.9826 support the selection of the quadratic model for the generation of polynomial equations. The adequate precision of 55.1604 indicates an adequate signal to navigate the design space. As per the ANOVA results, Response IV was significantly affected by B, C, and B^2^ factors via synergistic effect (*p* value < 0.0001) [39,40] with the highest magnitude of 38.46 for factor A (Table 4). Factors A and AB affect the response antagonistically.

Inhibition zone = +16.09 − 0.7066 A + 9.67 B + 2.21 C − 0.0588 AB − 0.0588 AC + 0.6912 BC − 0.1125 A^2^ − 1.49 B^2^ + 0.5583 C^2^ (7)

Serum Creatinine = +0.1198 − 0.0053 A + 0.0236 B + 0.0020 C − 0.0019 AB − 0.0009 AC + 0.0006 BC + 0.0003 A^2^ + 0.0079 B^2^ + 0.0007 C^2^ (8)

**Figure 2:**
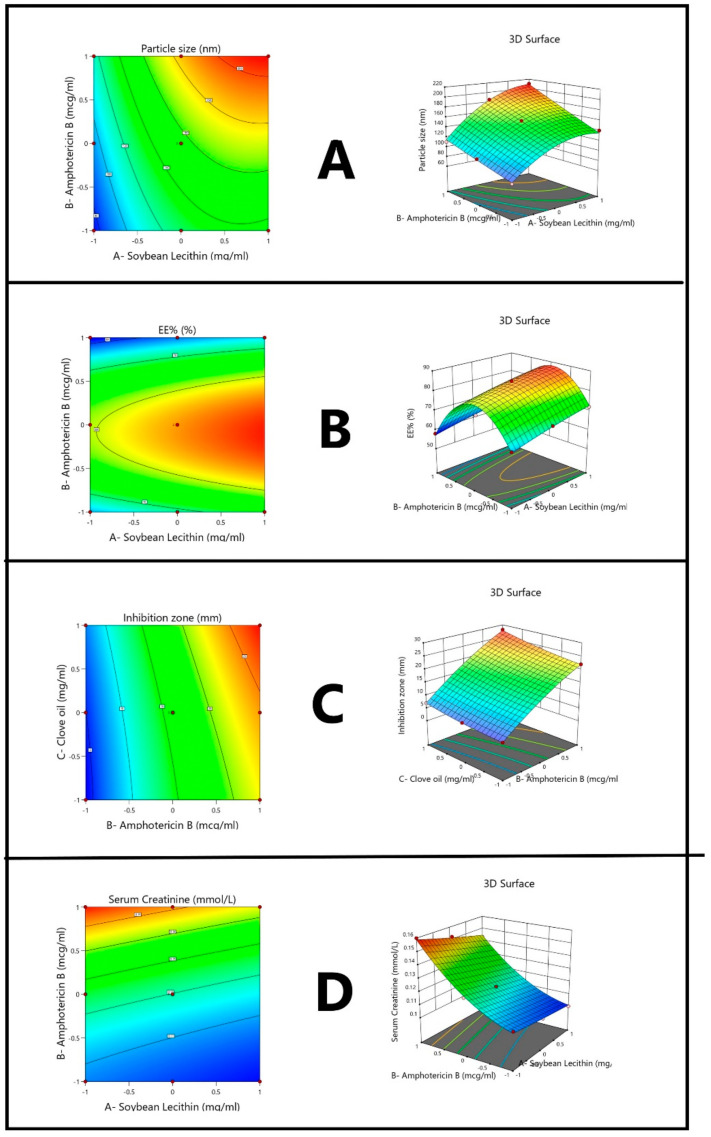
Contour and 3-dimensional response surface graphs of (**A**) particle size, (**B**) entrapment efficacy, (**C**) inhibition zone, and (**D**) serum creatinine.

RSM was applied to analyze the impact of these selected factors, and the respective contour plots and 3-dimensional graphs are shown in Figure 2. The results confirm the significant effect of the selected variables on particle size. Experimental runs with a smaller soybean concentration resulted in the formation of smaller-sized particles, which is in agreement with previous research [44]. The surfactant hydrophilic-lipophilic (HLB) value in the range of 12–16 is considered ideal for the production of nanotransferosomes. In contrast, HLB of 6–11 in soybean favors the formation of nano-drug delivery systems with smaller particle size. This is because smaller particles can more effectively reach the target tissues through systemic circulation and easily overcome the barrier problems associated with intranasal delivery. Conversely, the distribution of moderate size particles can lessen the risk of embolism. Moreover, higher levels of soybean concentration can enhance the entrapment efficacy and inhibition zone, further influencing the serum creatinine levels in an antagonistic way. According to the results obtained, the soybean and AMP concentrations largely impacted all the selected responses but in different ways [45]. Specifically, particle size, inhibition zone, serum creatinine, and EE were affected by AMP in a synergistic and antagonistic manner. As stated in literature, low oil levels can increase the particle size since the coalescence of oil droplets and added surfactant concentration can overcome the permeation problem. Conversely, increasing the proportion of surfactant in the nanotransfersomes can help in particle size reduction, while clove oil synergistically affects all other responses.

**Figure 3:**
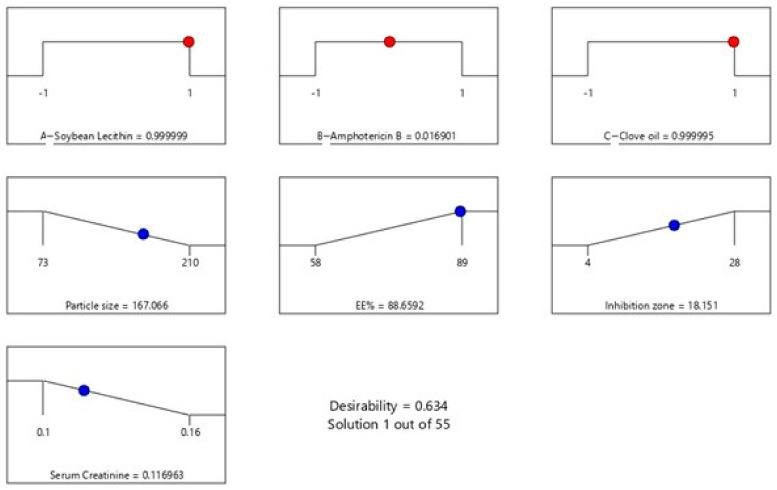
Desirability graph for optimization of AMP-NT.

Lastly, the formulation of AMP-NT was optimized by setting the desired goals for each response and simultaneously applying the global desirability function (D). On the basis of these criteria, the desirability plot was generated with a D value of 0.634 (Figure 3). In conclusion, the optimized formulation was determined to be 300 mg/mL soybean lecithin, 200 mg/mL amphotericin B, and 150 mg/mL clove oil, resulting in a particle size of 155.09 nm, 84.30% EE, inhibition zone of 16.0889 mm, and 0.1197 mmol/L serum creatinine. those results showed no significant difference with the predicted results shown in Figure 3. For example the actual value for serum creatinine is 0.1197 mmol/L while the predicted value in the figure is 0.1169 mmol/L, which indicated no significant difference between predicted and actual values.

### 3.6. Evaluation of AMP-NT In Situ Gels

The optimized formulation of NT was used to further prepare the AMP-NT in situ gels and evaluate various parameters. The results are depicted in Table 5. AMP-NT in situ gel had a clear appearance. The gelling capacity was found to be positive, and the quick gel transformation indicates the transformation of the formulation into gel was rapid for prepared in situ gel. A noticeable increase in the viscosity before and after gelation (2.93–34.26 cP) can be attributed to the presence of DCG in the formulation. However, this increased viscosity of the prepared formulation may limit its retention time in the nasal cavity since transformation into the gel may cause uneasiness in the nose. Comparatively, the expansion coefficient of the AMP-NT in situ gel was found to be 2.7%, indicating very slight expansion and, thus, minimal discomfort in patients [46]. The optimal gel should allow for easy administration but should not leak from the nasal cavity; hence, gel strength is a vital parameter to consider when formulating the in situ gel. Herein, the gel strength was determined to be 38.5 s, which is in the acceptable range of 25–50 s. Gel strength less than 25 s can lead to rapid erosion, and that greater than 50 s can cause much discomfort.

### 3.7. In Vitro Release

**Figure 4:**
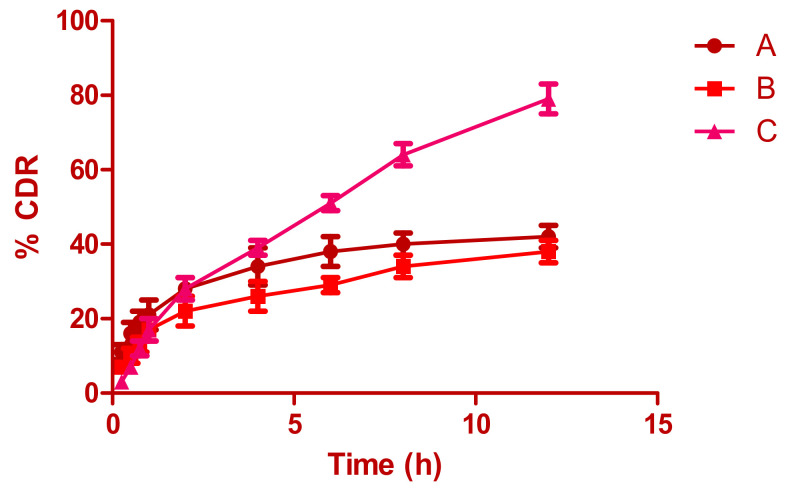
In vitro drug release profile from (A) amphotericin B released from aqueous suspension, (B) amphotericin B released from in situ gel loaded with drug aqueous suspension, and (C) amphotericin B released from optimized nanotransferosomal in situ gel.

Figure 4 shows the drug-release profile from the aqueous suspension and optimized AMP-NT in situ gel. Incomplete drug release (79.25% AMP) was observed in both the aqueous suspension and AMP in situ gel even at the end of 12 h, which may be due to the incomplete absorption of AMP in the selected formulations. Enhanced drug absorption was observed from nanotransferosomes, primarily credited to the reduced particle size. According to the inflection points observed in the release profile, the initial drug release was rapid due to incomplete gel formation. Thereafter, gelation further caused slow drug release from the in situ gel and reached a steady state concentration with remaining formulations.

### 3.8. Ex Vivo Permeation

Ex vivo permeation results of the aqueous suspension, optimized formula, and optimized formula prepared without clove oil through nasal membrane are presented in Table 6. A significant difference between the evaluated parameters was observed in all formulations.

In the optimized formulation with clove oil, 79.45% AMP permeated through the nasal membrane in accordance with the dissolution results, while only 37.2% permeated in the optimized formulation without clove oil, confirming the importance of clove oil.

Moreover, the prepared AMP-NT has the potential to penetrate and be retained in the nasal tissue, thus altering the permeation pathways of lipids. Moreover, the hydrophobic part of AMP-NT can further hydrate the internal area of the nasal cavity and plays a vital role in the uptake of the drug loaded by the tissue [47].

### 3.9. Nephrotoxicity Studies

Table 7 presents the various renal function parameters that were measured from rabbit plasma treated with amphotericin B aqueous suspension and the optimized amphotericin B optimized nanotransferosomal in situ gel against the control group. The results of the group treated with amphotericin B aqueous suspension indicated significant changes nearly in all measured responses compared to the control group. While the results of the group treated with amphotericin B optimized nanotransferosomal in situ gel showed no significant difference in all measured responses compared to the control group

The glomerular filtration rate is used to measure the excretion of a drug in urine [48]. In the current study, the large glomerular rate indicates that a large concentration of the optimized formulation was found in the urine.

Moreover, a decrease in the serum calcium levels in the test samples may be attributed to hypoproteinaemia, while a slight increase in sodium and potassium levels was observed with the optimized AMP-NT in situ gel.

## 4. Conclusions

In the present study, an AMP-NT in situ gel was prepared to study its effect against *Aspergillus flavus* in the nasal cavity. A Box–Behnken design, ANOVA, and polynomial equations were applied to optimize the concentrations of various parameters by statistical methodology. The optimized formulation was determined to be 300 mg/mL soybean lecithin, 200 mg/mL amphotericin B, and 100 mg/mL clove oil, which further achieved a minimum particle size and serum creatinine levels and maximum EE and zone of inhibition. The optimized AMP-NT was further prepared into an in situ gel system, optimized for suitable intranasal delivery, and evaluated for various in vitro, ex vivo, and in vivo parameters. In vitro, ex vivo permeation, and nephrotoxic studies confirm the enhanced percentage of drug permeation into the nasal tissues, owing to the presence of clove oil.

## Figures and Tables

**Table 1:** Experimental plan for amphotericin B-loaded nanotransferosomal formulations (AMP-NT).

Independent Variables	Levels			Dependent Variables	Constraints
	−1	0	+1		
Soybean Lecithin (mcg/mL) − X_1_	100	200	300	Particle Size (nm)	Minimum
Amphotericin B (mcg/mL) − X_2_	100	200	300	*EE*(%) Inhibition Zone	Maximum Maximum
Clove oil (mcg/mL) − X_3_	50	100	150	Serum creatinine	Minimum

*EE*%: Entrapement Effeciency.

**Table 2:** Projected trail formulations and their observed responses as per Box–Behnken design.

Run	A: Soybean Lecithin	B: Amphotericin B	C: Clove Oil	Particle Size	EE%	Inhibition Zone	Serum Creatinine
	mg/mL	mcg/mL	mg/mL	nm	%	mm	mmol/L
1	0	−1	1	120	70	7	0.106
2	0	−1	−1	130	67	4	0.102
3	0	1	−1	190	60	22	0.149
4	−1	1	0	110	58	25	0.16
5	−1	0	1	91	81	19	0.128
6	1	0	−1	170	87	14	0.116
7	1	1	0	210	65	23	0.143
8	−1	0	−1	100	77	15	0.122
9	−1	−1	0	73	64	6	0.109
10	0	0	0	154	85	16	0.12
11	0	1	1	179	63	28	0.155
12	1	−1	0	140	72	4	0.1
13	1	0	1	164	89	18	0.118
14	0	0	0	156	84	16	0.12
15	−1	−1	−1	80	61	4	0.107
16	−1	0	0	96	79	17	0.124
17	0	1	0	185	61	24	0.152
18	0	−1	0	126	69	5	0.104
19	1	1	1	204	66	27	0.146

**Table 3:** Fit summary of responses.

	PS		EE		Inhibition Zone		Serum Creatinine	
Source	Adjusted R^2^	Predicted R^2^	Adjusted R^2^	Predicted R^2^	Adjusted R^2^	Predicted R^2^	Adjusted R^2^	Predicted R^2^
Linear	0.9070	0.8700	−0.0540	−0.4099	0.9859	0.9799	0.9511	0.9364
2FI	0.8931	0.7941	−0.2386	−1.2717	0.9846	0.9692	0.9416	0.8989
**Quadratic**	**0.9936**	**0.9715**	**0.9967**	**0.9911**	**0.9978**	**0.9935**	**0.9946**	**0.9826**
Cubic	0.9995		0.9977		0.9994		0.9996	

PS: Particle size; EE: Entrapement Effecieny.

**Table 4:** Analysis of variance (ANOVA) results for three responses.

Term	Responses							
	PS		Steady State flux (JSS)		Inhibition Zone		Serum Creatinine	
	F-Value	*p* Value	F-Value	*p* Value	F-Value	*p* Value	F-Value	*p* Value
Model	310.83	<0.0001	598.28	<0.0001	915.93	<0.0001	367.49	<0.0001
A-Soybean Lecithin	1293.11	<0.0001	536.19	<0.0001	33.64	0.0003	136.07	<0.0001
B-Amphotericin B	790.91	<0.0001	401.96	<0.0001	7044.42	<0.0001	3033.65	<0.0001
C-Clove oil	16.99	0.0026	66.37	<0.0001	304.34	<0.0001	18.60	0.0020
AB	29.26	0.0004	1.10	0.3224	0.1094	0.7484	8.47	0.0173
AC	0.3638	0.5613	3.94	0.0785	0.1094	0.7484	1.96	0.1952
BC	0.0014	0.9708	0.0119	0.9155	15.12	0.0037	0.7506	0.4088
A^2^	174.48	<0.0001	4.27	0.0688	0.3428	0.5726	0.2112	0.6568
B^2^	0.1513	0.7063	4141.47	<0.0001	56.84	<0.0001	113.94	<0.0001
C^2^	0.5316	0.4845						
Lack of Fit	6.40	0.2969	0.6371	0.7544				

**Table 5:** Evaluation of optimized AMP-NT in situ gel.

Formulation	Parameter	Result
AMP-NT in situ gel	Gelling Capacity	+ve
	CIC%	0.17
	S%	2.7
	Viscosity before gellation (cp)	2.93 ± 0.37
	Viscosity after gellation (cp)	34.26 ± 3.01
	Gel strength	38.5 s

CIC%: Critical Ionic Concentration; S%: Expansion Coeffecient.

**Table 6:** Ex vivo permeation results.

Permeation Parameters	Optimized Formula	Optimized Formula Prepared without Clove Oil	Aqueous Suspension
Cumulative amount permeated (μg/cm^2^)	8915 ± 808	4822 ± 406	1813 ± 152
Cumulative percent permeated	79.45%	37.2%	
Steady state flux, Jss, (μg/cm^2^.min)	4.933 ± 1.02	3.011 ± 1.11	1.254 ± 0.31
Permeability coefficient, P, (cm/min)	3.101 × 10^−3^	1.411 × 10^−3^	0.411 × 10^−3^
Diffusion coefficient, D, (cm^2^/min)	16.22 × 10^−5^	11.18 × 10^−5^	5.66 × 10^−5^
Enhancement factor (EF)	5.562	3.013	------

**Table 7:** Various renal functional parameters evaluated from rabbit plasma for control and samples administered.

	Parameters	Glucose (mmol/L)	Creatinine (mmol/L)	Urea (mmol/L)	Calcium (mmol/L)	Sodium (mmol/L)	Potassium (mmol/L)
Control group	Day 1st	6.1 ± 0.4	0.1 ± 0.01	6.5 ± 1.1	3.4 ± 0.2	139 ± 3	3.8 ± 0.3
	Day 5th	6.4 ± 0.2	0.1 ± 0.01	6.3 ± 0.9	3.3 ± 0.2	137 ± 2	3.8 ± 0.4
	Day 10th	6.3 ± 0.3	0.11 ± 0.01	6.9 ± 0.6	3.3 ± 0.3	141 ± 5	3.7 ± 0.3
Amphotericin B aqueous suspension group	Day 1st	6.1 ± 0.4	0.1 ± 0.01	6.3 ± 0.4	3.2 ± 0.2	138 ± 6	3.8 ± 0.2
	Day 5th	5.4 ± 0.2 *	0.13 ± 0.01 *	6.1 ± 0.3	2.88 ± 0.2	147 ± 4 *	4.2 ± 0.2
	Day 10th	4.95 ± 0.1 *	0.16 ± 0.02 *	5.2 ± 0.3 *	2.11 ± 0.4 *	159 ± 4 *	4.8 ± 0.3 *
Amphotericin B optimized nanotransferosomal in situ gel group	Day 1st	6.1 ± 0.3	0.1 ± 0.01	6.4 ± 0.3	3.3 ± 0.3	138 ± 4	3.8 ± 0.2
	Day 5th	6.2 ± 0.1	0.11 ± 0.01	6.3 ± 0.1	3.0 ± 0.2	141 ± 3	4.0 ± 0.2
	Day 10th	6.05 ± 0.1	0.12 ± 0.01	6.3 ± 0.2	2.9 ± 0.2	144 ± 3	4.1 ± 0.3

(*) *p* value < 0.05 indicates a significant difference between parameters in comparison with the control group.

## Data Availability

Not applicable.
